# Electrospun Azithromycin-Laden Gelatin Methacryloyl Fibers for Endodontic Infection Control

**DOI:** 10.3390/ijms232213761

**Published:** 2022-11-09

**Authors:** Afzan A. Ayoub, Abdel H. Mahmoud, Juliana S. Ribeiro, Arwa Daghrery, Jinping Xu, J. Christopher Fenno, Anna Schwendeman, Hajime Sasaki, Renan Dal-Fabbro, Marco C. Bottino

**Affiliations:** 1 Department of Cariology, Restorative Sciences, and Endodontics, School of Dentistry, University of Michigan, Ann Arbor, MI 48103, USA; 2 Comprehensive Care Centre of Studies, Faculty of Dentistry, Universiti Teknologi MARA (UiTM), UiTM Campus Sg Buloh, Jalan Hospital, Sungai Buloh 47000, Malaysia; 3 Department of Clinical Dentistry (Endodontics), School of Dentistry, Federal University of Santa Catarina, Florianopolis 88040-900, Brazil; 4 Department of Restorative Dental Sciences, School of Dentistry, Jazan University, Jazan 45142, Saudi Arabia; 5 Department of Biologic and Materials Sciences & Prosthodontics, University of Michigan School of Dentistry, Ann Arbor, MI 48109, USA; 6 Department of Pharmaceutical Sciences, College of Pharmacy and Biointerfaces Institute, University of Michigan, Ann Arbor, MI 48103, USA; 7 Department of Biomedical Engineering, College of Engineering, University of Michigan, Ann Arbor, MI 48103, USA

**Keywords:** antimicrobial, electrospinning, dentistry, drug delivery, endodontics, fibers

## Abstract

This study was aimed at engineering photocrosslinkable azithromycin (AZ)-laden gelatin methacryloyl fibers via electrospinning to serve as a localized and biodegradable drug delivery system for endodontic infection control. AZ at three distinct amounts was mixed with solubilized gelatin methacryloyl and the photoinitiator to obtain the following fibers: GelMA+5%AZ, GelMA+10%AZ, and GelMA+15%AZ. Fiber morphology, diameter, AZ incorporation, mechanical properties, degradation profile, and antimicrobial action against *Aggregatibacter actinomycetemcomitans* and *Actinomyces naeslundii* were also studied. In vitro compatibility with human-derived dental pulp stem cells and inflammatory response in vivo using a subcutaneous rat model were also determined. A bead-free fibrous microstructure with interconnected pores was observed for all groups. GelMA and GelMA+10%AZ had the highest fiber diameter means. The tensile strength of the GelMA-based fibers was reduced upon AZ addition. A similar pattern was observed for the degradation profile in vitro. GelMA+15%AZ fibers led to the highest bacterial inhibition. The presence of AZ, regardless of the concentration, did not pose significant toxicity. In vivo findings indicated higher blood vessel formation, mild inflammation, and mature and thick well-oriented collagen fibers interweaving with the engineered fibers. Altogether, AZ-laden photocrosslinkable GelMA fibers had adequate mechanical and degradation properties, with 15%AZ displaying significant antimicrobial activity without compromising biocompatibility.

## 1. Introduction

Dental trauma and caries are the most prevalent causes of pulp necrosis, leading to tooth loss if left untreated [1]. It is well recognized that pulpal necrosis in permanent teeth with incomplete apex development presents a severe danger of fracture and therefore decreases the long-term survival [2,3]. To diminish this issue, the indication of the once termed revascularization technique to treat necrotic immature teeth has been documented during the last decade [4]. Briefly, this two-step regenerative-based technique first includes disinfecting the root canal system with irrigation solutions and minimal instrumentation, then placing an intracanal medication containing antibiotics to eliminate and restrict bacterial growth to improve the regenerative outcomes [5,6,7,8].

Although regenerative endodontics constitutes a fairly modern clinical domain and has matured over the last two decades, the topic still presents unstudied potential therapeutics for immature permanent teeth with pulp necrosis [9]. For instance, recent advances in electrospinning technology have made the synthesis of natural and/or synthetic polymer scaffolds and drug delivery systems feasible, with significant clinical potential to manage endodontic infection prior to regenerative procedures [7,10,11].

Gelatin methacryloyl (GelMA) is a semi-synthetic biocompatible, degradable, and tunable hydrogel that mimics many essential characteristics of the native extracellular matrix (ECM). Furthermore, GelMA contains cell binding and matrix metalloproteinase (MMP)-sensitive degradation sites and has shown great potential in drug delivery and regenerative medicine [12,13]. From a chemical standpoint, GelMA is synthesized by replacing the amine-containing side groups of gelatins with methacrylamide and methacrylate groups [14,15]. Importantly, due to its hydrophilicity, GelMA can be incorporated with distinct compounds, including but not limited to antibiotics and other clinically relevant therapeutic agents (e.g., dexamethasone-loaded clay nanotubes) [14,15,16]. Notably, when combined with a photoinitiator, customarily 2-Hydroxy-1-(4-(2-hydroxyethoxy)phenyl)-2-methylpropan-1-one (Irgacure^TM^ 2959) and ultraviolet (UV) light with appropriate intensity and wavelength, the GelMA precursor undergoes radical polymerization to generate a covalently crosslinked fibers network [12]. Regrettably, the use of UV light has been proven to evoke several biological deleterious effects, such as DNA damage by pyrimidine dimerization or the production of reactive oxygen species (ROS), leading to oxidative damage to DNA, accelerated tissue aging, and immunosuppression [17]. Nonetheless, it has been recently established that when using a proper solvent, GelMA can be employed into the fabrication of photocrosslinkable (UV) nanofibers [15]; however, aside from the stated phototoxic effects, UV light penetrates tissues, hydrogels, and fibers only to a limited extent, posing a further barrier to its medical uses [12]. Collectively, these issues have prompted scientists to examine the use of higher-wavelength light, such as visible light, combined with a photoinitiator that can be triggered at those wavelengths [12]. In this way, lithium phenyl(2,4,6-trimethylbenzoyl)phosphinate (LAP), a colorless, single-component initiation system with strong thermal stability and good solubility, which can be triggered by conventional dental curing light, has garnered substantial notice [18].

Azithromycin (AZ) is a semi-synthetic, acid-stable second-generation macrolide antibiotic with a 15-membered azlactone ring, demonstrating a broad spectrum of bacteriostatic action and enhanced pharmacokinetics, and is clinically proven to be effective against Gram-positive, Gram-negative, and atypical infections [19]. Interestingly, in a recent study assessing the effect of AZ on pre-existing experimental periapical lesions, the antibiotic-modulated macrophage polarization from pro-inflammatory (M1) macrophages to pro-resolving (M2) macrophages led to the resolution of periapical inflammation through its immunomodulatory effect [20].

In the present study, we detail for the first time the development of visible-light photocrosslinkable GelMA electrospun fibers loaded with azithromycin as a biodegradable and biocompatible localized drug delivery system for endodontic infection control. Herein, we hypothesized that a combination of LAP-triggered photocrosslinked GelMA fibers with optimal azithromycin content would lead to significant antimicrobial action against endodontic pathogens, with acceptable toxicity to dental pulp stem cells and minimal inflammatory effects upon subcutaneous implantation of the engineered AZ-laden GelMA fibers in rats.

## 2. Results

### 2.1. Morphological and Chemical Characteristics

**Figure 1:**
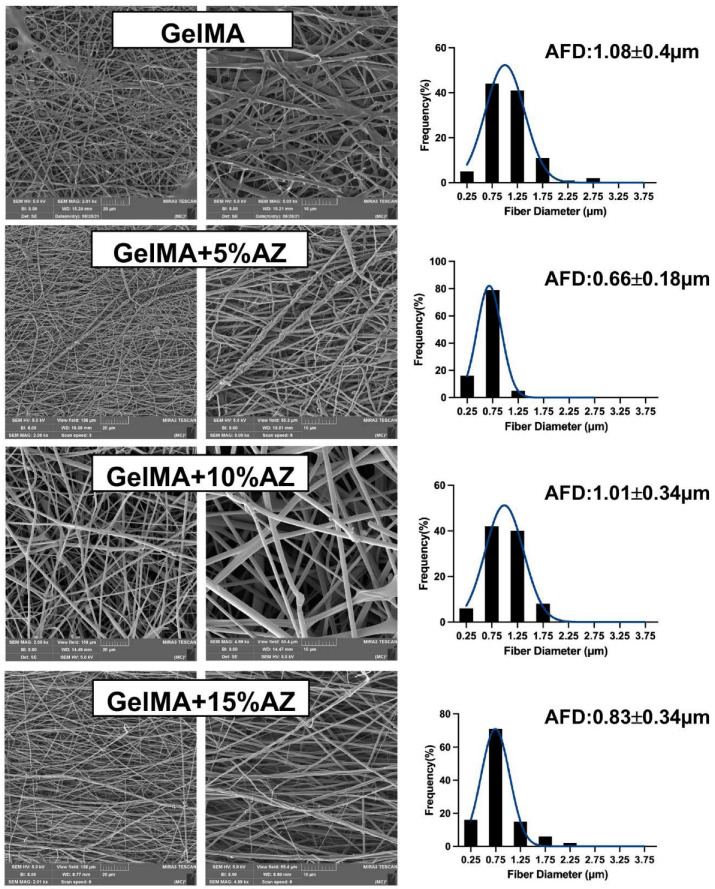
(**Left**) Representative scanning electron microscopy (SEM) images of the electrospun azithromycin-laden gelatin methacryloyl and the pure (antibiotic-free) GelMA fibers. (**Right**) Histograms showing the fiber diameter frequency and average fiber diameter (AFD) with standard deviation.

A bead-free fibrous network with interconnected pores was observed for all groups (Figure 1). The diameters of GelMA+5%AZ and GelMA+15%AZ electrospun fibers ranged between 0.66 ± 0.18 µm and 0.83 ± 0.34 µm and were considerably smaller (*p* < 0.05) than pure GelMA fibers (1.08 ± 0.40 µm). At the same time, the fiber diameter of the GelMA+10%AZ fibers (1.01 ± 0.34 µm) did not differ from the control group (*p* < 0.05).

**Figure 2:**
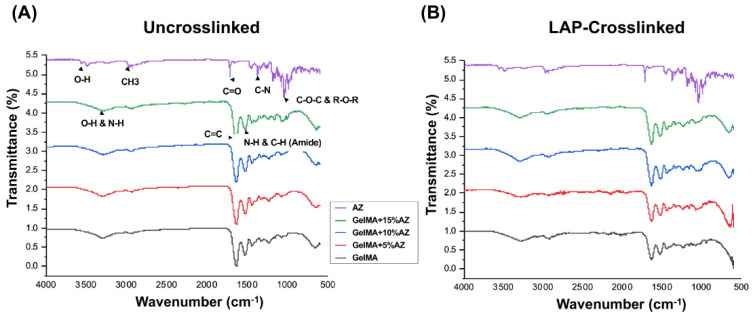
Fourier transform infrared spectroscopy (FTIR) spectra of (**A**) uncrosslinked and (**B**) crosslinked GelMA-based fibers.

The FTIR analysis and interpretations of peaks for pure GelMA and different AZ concentrations before and after photocrosslinking are presented in Figure 2. The characteristic peaks of AZ at 3493.03, 1719.9, 1249.85, and 1082.15 cm^−1^ correspond to the hydroxyl group, H-bonded OH, ketone carbonyl compound, aromatic ethers, aryl -O stretch, and organic sulfates [21]. All four similar characteristic peaks were present in uncrosslinked fibers; 3285.51–3307.80, 1645.29–1650.39, 1242.73–1243.73, and 1080.57–10.80.92 cm^−1^. The spectrum of AZ has characteristic peaks at 2907, 1720, 1363, 1190, 1108 cm^−1^, and about 1350 corresponded, respectively, to C-H (methyl group) stretching, C=O stretching, C=C bending, C-O-C asymmetrical stretching, C-O-C symmetrical stretching, and C-N stretching.

Regarding pure GelMA fibers, peaks at 3288 cm^−1^,1640 cm^−1^, 1650 cm^−1^, and 1530 cm^−1^ correspond to O-H and N-H stretching, C=C stretching of the methacrylate group, C=O stretching of the amide group, and N-H bending coupled to C-H stretching of the amide group, respectively. Peaks for AZ between 100–1300 cm^−1^ are present for the GelMA+AZ groups, particularly for crosslinked groups. Conversely, azithromycin’s main peak at 1720 cm^−1^ is unclear in the GelMA+AZ groups, possibly because of the chemical reaction between the methyl group of AZ (CH_3_) and the methacrylate group of GelMA (C=C).

### 2.2. Mechanical Properties and Degradation Profile

**Figure 3:**
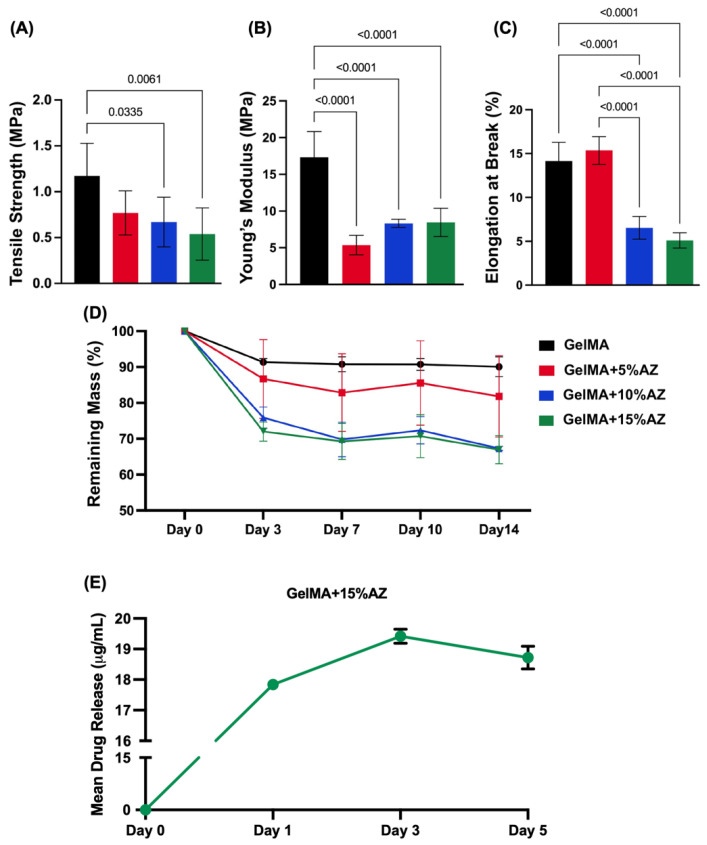
Mechanical analyses of the engineered GelMA-based electrospun fibers laden with 3 distinct amounts of azithromycin. (**A**) Tensile strength in MPa; (**B**) Young’s modulus in MPa; (**C**) Elongation at break in %; and (**D**) Degradation profile over 14 days in %. (**E**) Release profile of AZ from GelMA+15%AZ fibers over 5 days. Data are shown as mean and standard deviation.

The tensile strength of the electrospun samples (in MPa) decreased, along with the ascending concentration of the added AZ as follows: 1.17 ± 0.35 (GelMA), 0.76 ± 0.24 (GelMA+5%AZ), 0.66 ± 0.27 (GelMA+10%AZ), and 0.53 ± 0.28 (GelMA+15%AZ). The tensile strength of the GelMA fibers was significantly (*p* < 0.05) higher than GelMA+10%AZ and GelMA+15%AZ. Meanwhile, all the AZ-laden GelMA fiber groups did not show significant differences in strength between them (Figure 3A). Young’s modulus was significantly reduced by adding AZ to the GelMA fibers. However, no differences were found comparing the three distinct AZ concentrations (Figure 3B). When the elongation at break was evaluated, pure GelMA and GelMA+5%AZ exhibited similar values and were significantly (*p* < 0.05) higher than GelMA+10%AZ or GelMA+15%AZ (Figure 3C). The degradation profile for the synthesized GelMA-based fibers is shown in Figure 3D. All the groups had a higher mass loss in the first three days of PBS incubation. The greater degradability values were associated with groups loaded with higher AZ concentrations (*p* < 0.0001). Pure GelMA lost ~5% of mass by day 3, and the GelMA+15%AZ fibers lost about 33% of the mass, followed by 25% and 12% mass loss for 10% and 5% AZ-laden GelMA fibers, respectively. It is important to note that all groups remained stable after reaching a plateau from day 3 until day 14 with minimal mass loss variation.

### 2.3. Drug Release

The in vitro release profile of azithromycin from the GelMA+15%AZ fibers is shown in Figure 3E. A burst release of AZ was observed in the first 24 h, reaching 17.8 ± 0.04 μg/mL. From day 1 to day 3, an 8% increase in the antibiotic release was noticed, attaining 19.4 ± 0.23 μg/mL. From day 3 to day 5, there was a slight decrease in the AZ release compared to day 3, achieving 18.72 ± 0.36 μg/mL.

### 2.4. Cytocompatibility

**Figure 4:**
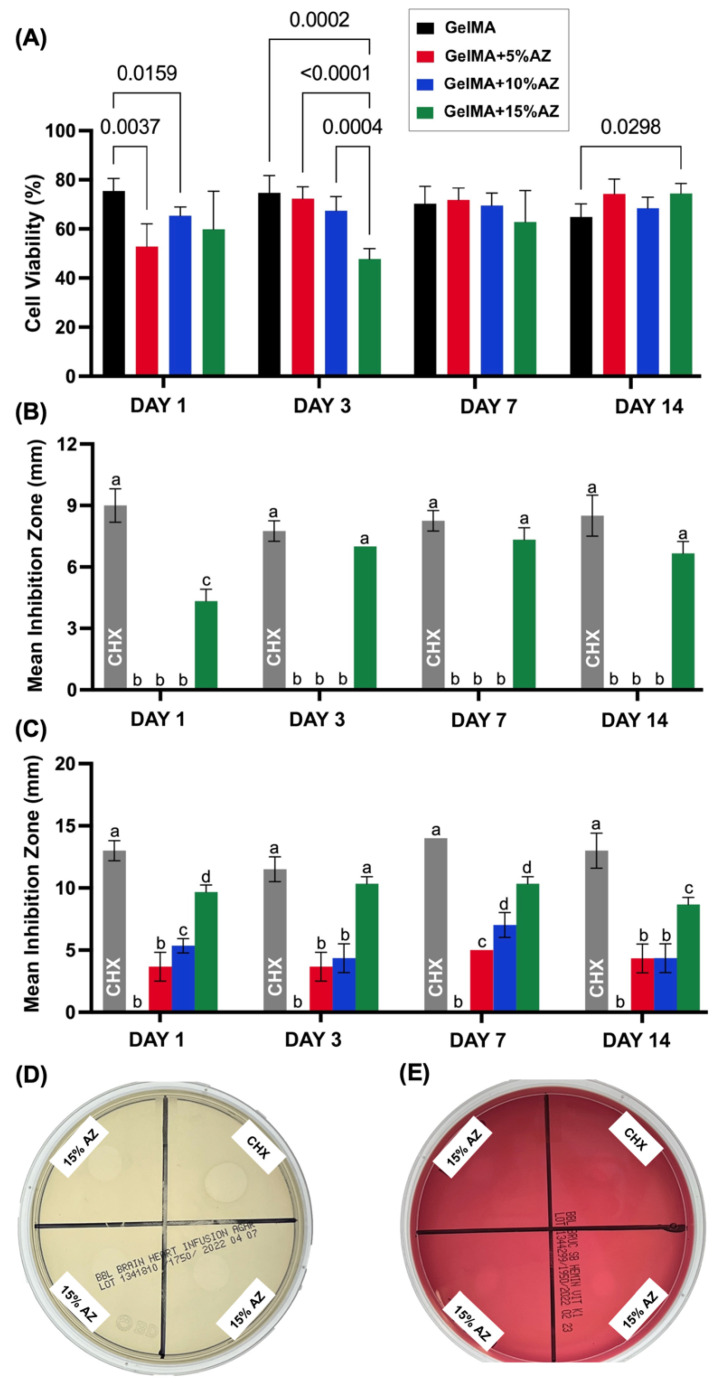
(**A**) Graphic representation of mean and standard deviation of hDPSCs cell viability (%) determined after 72 h using aliquots collected at 1, 3, 7, and 14 d. The percentage of cell viability was normalized by the mean absorbance of hDPSCs cultured on the plate on day 1 (100%). Absorbance was measured at 490 nm. Antimicrobial potential against *Actinomyces naeslundii* (**B**), and *Aggregatibacter actinomycetemcomitans* (**C**), evaluated through the Kirby-Bauer diffusion test (**D**) for *Aa* and (**E**) for *An* using 0.12% CHX as a positive control. Different lowercase letters represent statistical differences between groups compared on the same day (*p* < 0.05).

Determining the in vitro cytotoxicity of our novel GelMA-based fibers is essential to supporting its potential application as a localized drug delivery system for endodontic infection control. Here, we investigated the cytocompatibility of the fabricated AZ-laden GelMA-based fibers with hDPSCs. The results are shown in Figure 4A as the viability percentage of each group using the control group (cells seeded but without any aliquots) as 100%. Compared with the pure GelMA group, the number of surviving cells decreased after exposure to the aliquots collected on day 1 for AZ concentrations at 5 and 10%. The same pattern was observed for aliquots collected on day 3, with the 15% AZ group showing the highest decrease (*p* = 0.0002), which was significantly distinct from 10% AZ (*p* = 0.0004). For aliquots collected on day 7, a viability plateau of around 70% was reached for all tested groups, without differences. For aliquots collected on day 14, the AZ-laden fibers demonstrated slightly higher viability than pure GelMA, with the GelMA+15%AZ group displaying significant (*p* = 0.0298) differences.

### 2.5. Antimicrobial Assessment

The electrospun AZ-laden GelMA-based fibers’ antimicrobial effect was assessed through agar diffusion against *Actinomyces naeslundii* and *Aggregatibacter actinomycetemcomitans* performed by an indirect contact assay (i.e., eluates collected over 14 days from sample incubation in PBS). No inhibition was observed in the negative control (PBS). A 12 mm mean inhibition zone was observed for the positive control (0.12% chlorhexidine, CHX) for all time points. Agar diffusion analyses confirmed the antimicrobial properties of the AZ-laden GelMA fibers (Figure 4B–E). Only the highest AZ concentration (15%) had an antimicrobial effect against *An* on all aliquots collected from days 1 to 14 (Figure 4B). For the *Aa*, GelMA fibers containing 15% AZ presented a higher inhibition (~10 mm) than 10% and 5% AZ concentrations for aliquots collected on days 1 and 3. Notably, GelMA+15%AZ aliquot on day 3 led to similar inhibition when compared to the positive control (Figure 4C). For aliquots collected on day 7, 10% and 15% AZ concentrations demonstrated statistically higher inhibition than 5%; and for 14-day aliquots, antibiotic activity was still present, since the 15% AZ concentration demonstrated statistically higher inhibition than the other two AZ-laden groups (Figure 4C).

### 2.6. In Vivo Biocompatibility

**Figure 5:**
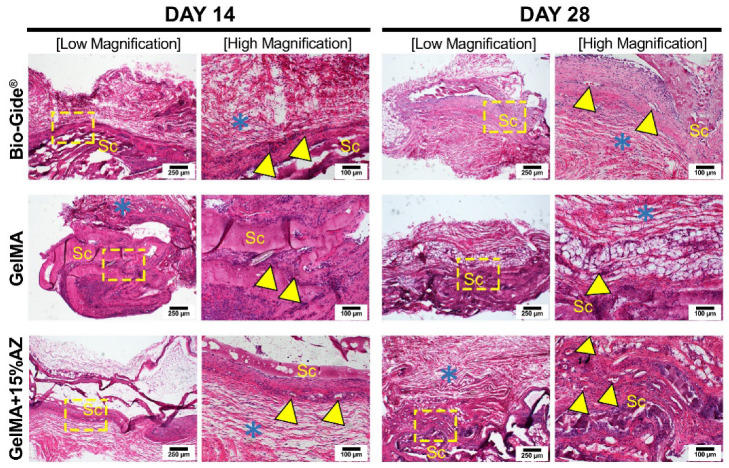
H&E-stained slices for implanted materials in rats’ subcutaneous tissue at 14 and 28 d. 4X magnification (scale bar = 250 μm) and 10X magnification (scale bar = 100 μm). A dashed yellow square delimits the area selected for higher magnification. Scaffold: Sc, Blood vessels: yellow arrowhead, Fibroblasts with collagen fibrils: blue asterisk. Note at 14 d, the enhanced inflammatory reaction evoked by pure GelMA, compared to the other two groups, and higher blood vessel formation and well-oriented collagen fibers from the group GelMA+15%AZ at 28 d.

**Figure 6:**
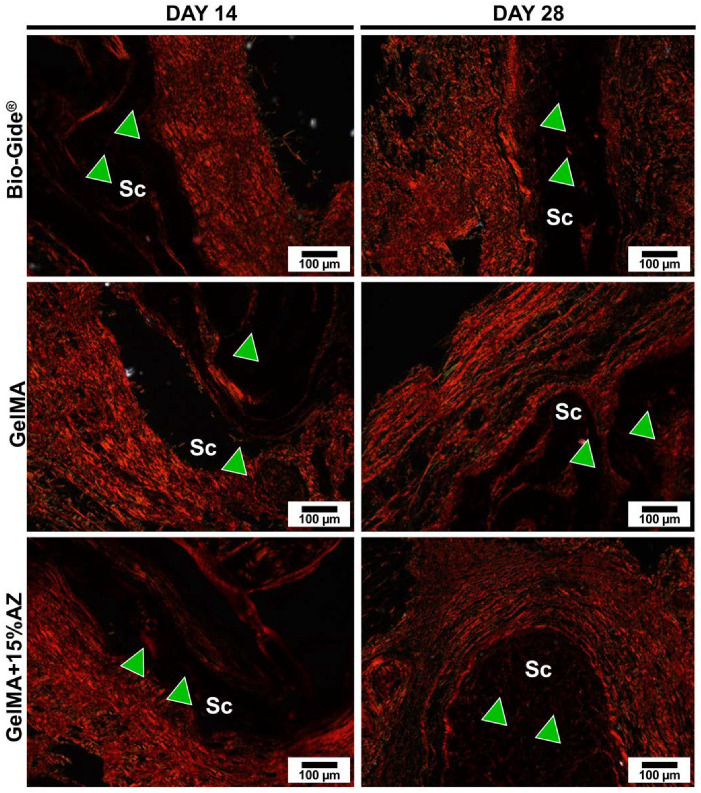
Representative Picrosirius Red stained slices for the implanted scaffolds in rat’s subcutaneous tissue at 14 days and 28 days. Original magnification: 10× (scale bar = 100 μm). Scaffold: Sc, Red fibers considered as mature and thick interspersing the scaffold: green arrowhead. On day 28, note the presence of mature collagen fibers interspersing the area initially occupied by the scaffold.

The biocompatibility (H&E) and collagen fiber production (Picrosirius Red) results of AZ-laden GelMA fibers subcutaneously implanted in rats are shown in Figure 5 and Figure 6. After 14 days, the host tissue response at the interface between the implanted material and subcutaneous tissue had an exacerbated inflammatory reaction for pure GelMA fibers when compared to Bio-Gide^®^ (control) and GelMA+15%AZ fibers mainly consisting of polymorphonuclear over the mononuclear inflammatory cells. Blood vessel formation was observed for the three groups, specifically for GelMA+15%AZ with larger diameters. At 28 days post-implantation, the inflammation for all 3 groups slightly decreased over time. Bio-Gide^®^ presented better results, with minimal mononuclear inflammatory infiltrate and initial material resorption. Pure GelMA evoked mild inflammation compared to the control, along with the presence of fatty infiltrates. The GelMA+15%AZ showed slightly decreased inflammation, predominantly consisted of mononuclear inflammatory cells, showing higher vascularity formation and well-organized collagen fibrils’ layers when compared to pure GelMA. There were no apparent differences in the level of degradation or fragmentation of the GelMA-based fibrous mats.

## 3. Discussion

Among the primary aims of endodontic treatment is to eradicate microbial infection, which is usually a multispecies infection involving aerobic and anaerobic bacteria, with obligate anaerobes being the predominant ones [22]. For this investigation, azithromycin was chosen based on a recently disclosed potential for eliminating pre-existing experimental periapical inflammation [20]. To the best of our knowledge, this is the first report on electrospun LAP-photocrosslinked GelMA-based fibers with and without the incorporation of azithromycin. Our findings indicate that incorporation of AZ into the GelMA matrix does not compromise biocompatibility and yet supports the development of antibiotic-laden GelMA-based fibers as a biodegradable and localized drug delivery method for endodontic therapy.

The GelMA-based fibers produced by electrospinning were observed to be homogeneous and smooth. The addition of AZ was observed to decrease the diameter for the 5% and 15% concentrations. This may be attributed to an enhancement of the electrical conductivity of the electrospinning solution. This higher conductivity suppresses varicose instability and enhances whipping instability, consequently forming finer fibers [23]. The novel GelMA-based fibers were also characterized based on their physicochemical characteristics. First, chemical analysis reveals that the incorporation of AZ did not affect the peaks related to vibrational bands of GelMA in the FTIR spectra, which supports the chemical stability of the fibers. Meanwhile, the AZ’s main peak at 1720 cm^−1^ is unclear in the GelMA+AZ groups, probably due to (i) the overlapping peaks of GelMA and AZ, and (ii) the chemical reaction between the methyl group of AZ (CH3) and the methacrylate group of GelMA; consequently, AZ-related peaks were not easy to identify. Nevertheless, the effective incorporation of AZ into GelMA nanofibers was confirmed based on the antimicrobial findings. Next, to demonstrate that the GelMA-based fibers can be successfully utilized as an effective and highly tunable platform for delivering antimicrobial drugs, an in vitro degradation assay was performed. Hydrogel degradation plays an essential role in the controlled drug release, avoiding rapid drug clearance, and leading to a long-term drug release [24]. Based on the presented results, it can be concluded that the degradation of AZ-laden fibers was directly proportional to the concentration of the added antibiotic. However, even with the highest concentration, after significant degradation in the first 24 h (around 30% weight loss), the fibers remained stable until day 14 (about 70% of the original weight). Currently, it is well-established that the use of GelMA at a concentration equal to or less than 15% (*w*/*v*) has a satisfactory degradation rate for tissue regeneration and is highly biocompatible [25]. In addition, the use of LAP has shown a direct influence on the rate of degradation, as well as on drug release [26]. Therefore, the ability to crosslink GelMA-based fibers ensured greater control of the degradation rate without compromising biocompatibility.

From a mechanical perspective, the presence of AZ led to a reduction in tensile strength, which was inversely proportional to the AZ concentration. Despite Young’s modulus being significantly reduced by AZ addition into the GelMA fibers, no differences were found comparing the three distinct concentrations. Notably, since the equilibrium between hydrophobicity and hydrophilicity is essential to producing physical-mechanically stable matrices, we hypothesized that the hydrophobicity of AZ led to the observed reduction in tensile strength, which based on the foreseen clinical applications as drug delivery systems (e.g., intracanal or in deep caries lesions) should not compromise the in vivo performance of the material.

Localized drug delivery systems represent a promising therapeutic strategy for applications in treating oral infections. An indispensable attribute of the present study was to validate the antimicrobial action of the AZ-laden GelMA fibers against endodontic pathogens associated with primary endodontic infection and persistent infection due to failed endodontic treatment [27,28,29]. In this case, our indirect agar diffusion antimicrobial results revealed that GelMA+15%AZ led to a substantial and prolonged antimicrobial action toward *An* and *Aa* due to the elevated antibiotic concentration, thus proving the release of AZ from the fibers from the first 24 h, keeping the antimicrobial action until day 14. Notably, based on our drug release investigation, the same pattern was observed; from the first day of incubation, the AZ released by the GelMA+15%AZ successfully attained higher values than 1 µg/mL, which represents the amount at which ≥90% of the *Aa* population is inhibited (MIC90) [30], and higher than the 0.5 µg/mL MIC for *An* [31]. Due to its dual-base structure, AZ is actively absorbed by various cells and acts by binding to the 23S rRNA of the bacterial 50S ribosomal subunit inhibiting the transpeptidation/translocation step of protein synthesis, resulting in the control of various bacterial infections. Specifically, AZ is known to accumulate inside neutrophils, enhancing phagocytic killing of the *Aa* [32].

In addition to the antimicrobial activity, assessing the in vitro cytotoxicity of the developed GelMA-based fibers is key to confirming their potential application and safety as an auxiliary drug delivery system for endodontic infection management. In the present study, using human dental pulp stem cells, a minor cytotoxic effect was observed for aliquots collected at days 1 and 3, likely due to the more significant release of AZ on the first 3 days. From day 7 and beyond, the incorporation of AZ did not compromise cell viability. Also, it is essential to note that the minor concentration of added LAP did not demonstrate cytotoxicity. These data reinforce safe use of the proposed nanofibers in endodontic infection control protocols and pulp regeneration therapies.

After the promising results from in vitro experiments, we decided to investigate the biocompatibility in vivo. The rat subcutaneous model explored cellular infiltration properties, collagen production, and GelMA-based fibers morphological changes. Although the implantation site diverged from the suggested clinical application, the subcutaneous in vivo biocompatibility test is a well-established model used to represent mechanisms and consequences of tissue-biomaterial interactions [33]. Hematoxylin/eosin, and picrosirius red images of retrieved tissue with implanted AZ-laden GelMA fibers indicated no compromised biocompatibility. Moreover, adding AZ to the newly synthesized GelMA-based fibers enhanced the collagen deposition between the fibers.

The anti-inflammatory properties of AZ can be attributed to its action on macrophages, since mechanistic studies demonstrated immunomodulatory activity through the regulation of cellular processes involved in inflammation through the reduction in NF-kB activation and, consequently, reduction in the up-regulation of pro-inflammatory cytokines [34,35]. Also, AZ exhibits immunomodulatory properties by shifting the inflammatory response toward an M2 macrophage state, characterized by regulation of inflammation and repair [36]. AZ also inhibits the expression of phospholipases A2, an enzyme involved in cell signaling processes that produces arachidonic acid byproducts [37], induced by LPS, an essential component of the outer membrane in Gram-negative bacteria, such as *Aggregatibacter actinomycetemcomitans* [38].

It is important to emphasize that, to the best of our knowledge, this is the first study reporting on the fabrication of visible-light photocrosslinkable GelMA-based fibers loaded with azithromycin as a biodegradable and biocompatible localized drug delivery system for endodontic infection control. Thus, more studies are needed to determine the drug release rate, stability, and durability of the antimicrobial activity. Lastly, since the healing of apical periodontitis is crucial for continuing root development and the overall regenerative outcome, we plan to address in a future investigation the potential benefits of AZ-laden GelMA electrospun fibers as alternative antimicrobial and immunomodulatory therapeutics within the context of regenerative endodontics.

## 4. Materials and Methods

### 4.1. Gelatin Methacryloyl Synthesis

GelMA production was performed following formerly reported studies [16,26]. Briefly, on a heating plate at 50 °C, type A gelatin from porcine skin (300 bloom, Sigma-Aldrich, St. Louis, MO, USA) at 10% *w*/*v* was solubilized into Dulbecco’s phosphate-buffered saline (DPBS, Gibco Invitrogen Corporation, Grand Island, NY, USA). Next, in a dropwise manner, 8 mL of methacrylic acid (MA) was added to the gelatin solution, allowing them to react for 2 h under continuous stirring. The reaction was interrupted by adding an equal quantity of DPBS at 40 °C. Lastly, unreacted monomers and salts were eliminated by dialyzing the mixture in deionized water (DI) and employing a 12–14 kDa dialyze tube at 45 ± 5 °C for 7 d, with DI water changed twice daily. The prepared solution was frozen at −80 °C, lyophilized (Labconco FreeZone 2.5L, Labconco Corporation, Kansas City, MO, USA) for a week, and stored at −80 °C until further use.

### 4.2. Electrospinning and Nanofibers Preparation

Pure GelMA (i.e., AZ-free) and AZ-laden GelMA fibers were engineered via electrospinning based on formulation of a single antibiotic at three distinct amounts (5%, 10%, and 15%, *w*/*w*), hereafter referred to as GelMA+5%AZ, GelMA+10%AZ, and GelMA+15%AZ, respectively. These formulations were based on an initial screening performed by our team to find suitable cytocompatibility and electrospinning ability. Briefly, lyophilized GelMA was dissolved at a concentration of 15% *w*/*v* in acetic acid (Sigma-Aldrich). The mixture was left on the hot plate at 50 °C overnight. Next, AZ was individually added at the specified concentrations and left stirring for 2 h. Then, a photoinitiator, i.e., lithium phenyl-2,4,6-trimethylbenzoylphosphinate (LAP, TCI America Inc., Portland, OR, USA), was added at the concentration 0.075% *w*/*v* to all groups [16,26]. The prepared GelMA-based solutions were separately loaded into 5 mL plastic syringes capped with a 27-gauge metallic needle. The electrospun fibers were spun using the specifications 2 mL/h, 18 cm distance, and 18 kV on a rotating mandrel overlaid with aluminum foil at 120 rpm in a custom-made electrospinning box. Notably, to protect the GelMA-based solutions from room light exposure during electrospinning, both the plastic syringes and the electrospinning box were wrapped with black paper. The electrospun mats were placed in a desiccator overnight at room temperature (RT) to ensure complete acetic acid evaporation. Lastly, the fibers were peeled from the aluminum foil, cut into the desired size according to the experiment, wetted with 100% amorphous ethanol, gently dried with low-lint wipes (Kimwipes, Kimberly-Clark Corporation, Irving, TX, USA), and light-cured for 5 min on each side using an LED light box (Light Zone II, BesQual-E300N, Meta Dental Corp, Glendale, NY, USA).

### 4.3. Morphological and Chemical Characteristics

Scanning electron microscopy (SEM, MIRA3, FEG-SEM, Tescan, Czech Republic) was performed to assess fiber morphology and microstructure. Using double-sided carbon adhesive tape, the samples were fixed on Al stubs and sputter-coated with a thin (~5–10 nm) layer of Au-Pd (SPI-Module Carbon/Sputter Coater, SPI Supplies, West Chester, PA, USA) before imaging. The average fiber diameter (AFD) was determined using ImageJ (National Institutes of Health, Bethesda, MD, USA) software. Four SEM images per group were used to measure fiber diameter and the data reported as average ± standard deviation (n = 25/image/group). To confirm AZ incorporation, Fourier-transform infrared spectroscopy FTIR (ATR-FTIR, Thermo-Nicolet IS-50, Thermo Fischer Scientific, Inc., Waltham, MA, USA) using attenuated total reflection was conducted. The spectra recording was performed between 4000 and 600 cm^−1^ at 4 cm^−1^ resolution.

### 4.4. Mechanical Properties

The tensile strength of the fibrous mats was evaluated by uniaxial tensile testing (eXpert 5601; ADMET Inc., Norwood, MA, USA) [39]. Testing of rectangular-shaped specimens (25 mm × 3 mm^2^, n = 6/group) was performed at a crosshead speed of 1 mm/min. Three distinct mechanical properties (i.e., tensile strength, Young’s modulus, and elongation at break) were recorded or determined from the load-position curves.

### 4.5. Degradation Profile

In vitro degradation of the fabricated GelMA-based fibers was assessed by incubating them in PBS and recording their weight variation over time. The electrospun fibrous mats were cut into square-shaped samples (20 × 20 mm), treated with 100% ethanol, and light-cured for 5 min/side. The samples (n = 3) were weighed, immersed into 2 mL sterile PBS, and incubated for 14 days. At prearranged time points, the samples were removed from the incubation medium, gently dried with low-lint wipes (Kimwipes, Kimberly Clark Corporation), and washed twice with DI water and dried at RT for 24 h before the weight was recorded. The degradation profile was calculated by the formula, where Wt denotes the residual weight over time and w0 denotes the original dried weight.

$$\mathrm{Degradation}\;\mathrm{ratio}\;\left(\%\right)=\frac{w_{t}}{w_{0}}\times 100$$

### 4.6. Drug Release

To determine the kinetics of azithromycin release, four square-shaped (10 × 10 mm^2^) GelMA+15%AZ fibrous mats were individually incubated in glass vials containing 10 mL PBS at 37 °C under constant shaking. After 1, 3, and 5 days, 1000 μL aliquots were drawn. Equal amounts of fresh PBS were added back to the incubation following aliquot retrieval. The AZ content was determined by quantifying the absorption of the clear supernatant using a UV-spectrophotometer (SpectraMax iD3, Molecular Devices LLC, San Jose, CA, USA) at 410 nm in triplicate. The AZ concentration at each time point was calculated by comparing it with the established standard curve [14].

### 4.7. Cytocompatibility

Four square-shaped (15 mm × 15 mm^2^) fibrous mats per group were individually incubated in glass vials containing Glucose Dulbecco Modified Eagle Medium (DMEM, Gibco Invitrogen Corporation) for up to 14 days. 500 μL aliquots were drawn at prearranged time points and identical amounts were added to maintain the extraction volume constant. Using a 0.22 μm nylon membrane under vacuum, the aliquots were filtered before further analysis [16].

Human dental pulp stem cells (hDPSCs) obtained from permanent third molars (passages 6–8) were cultured in DMEM containing 15% fetal bovine serum (FBS; Hyclone Laboratories, Inc., Logan, UT, USA) and 1% penicillin-streptomycin (Sigma-Aldrich) in a humidified incubator at 37 °C with 5% CO_2_. The cells were seeded at a density of 3 × 10^3^/well (100 µL cell suspension) in 96-well tissue culture microtiter plates. After 4 h of incubation, the media was removed and replaced by the collected aliquots in triplicate (100 µL), adjusted to 15%FBS and 1% penicillin-streptomycin, and the positive control (0.3% vol phenol solution) [16]. Quadruplicated wells were arranged using medium without cells (blank control) and medium with cells but without any aliquot (reflecting 100% survival) [16]. The microplates were then incubated in a 5% CO_2_ chamber. After 3 days, 20 µL CellTiter 96 Aqueous One Solution Reagent (Promega Corporation, Madison, WI, USA) was added to the test wells and incubated for 2 h. The color change reaction was assessed by reading the absorbance at 490 nm in a microplate reader (Spectra iD3; Molecular Devices LLC, San Jose, CA, USA) against blank wells [16].

### 4.8. Antimicrobial Assessment

GelMA-based AZ-laden fibers were further investigated using agar diffusion against *Actinomyces naeslundii* (*An*, American Type Culture Collection, ATCC 12104, Manassas, VA, USA), and *Aggregatibacter actinomycetemcomitans* (*Aa*, American Type Culture Collection, ATCC 43718). Like the procedure for cell compatibility, square-shaped (n = 3; 15 mm × 15 mm^2^) fibrous mats were first disinfected by UV light (30 min/side) and rinsed twice with sterile PBS. Next, the GelMA-based mats were individually incubated in PBS for 2 weeks. At predetermined time points, 500 μL aliquots were drawn and equal amounts were added to keep the extraction volume unchanged. The aliquots were stored at −20 °C until further use [26].

Broth cultures of bacterial strains were grown for 24 h at 37 °C in BHI broth (Becton, Dickinson & Co. Sparks, MD) in a 5% CO2 atmosphere (*A. actinomycetemcomitans*) or anaerobically in 5% CO_2_, 10% H_2_ in N_2_ atmosphere (*A. naeslundii*). Bacterial cultures on agar plates were grown under the same conditions, using tryptic soy agar with 5% *v*/*v* sheep blood (Hardy Diagnostics, Santa Maria, CA, USA) for *A. actinomycetemcomitans* and Brucella blood agar with hemin and vitamin K (Hardy Diagnostics) for *A. naeslundii*. One hundred µL of each broth culture was spread onto appropriate agar plates to create a lawn of bacteria. Four individual zones receiving 10 µL of eluates from AZ-laden GelMA-based fibers (days 1, 3, 7, and 14) were created on each plate. Chlorhexidine (0.12%) and sterile PBS were positive and negative controls, respectively. The plates were incubated according to the bacteria strain. After 2 days of incubation, the diameters (in mm) of the clear growth inhibition zones were calculated.

### 4.9. In Vivo Biocompatibility

All animal procedures followed the ARRIVE guidelines for reporting animal research and were in accordance with the procedures of the local Institutional Animal Care and Use Committee (PRO00010329). Four 6-week-old male Fischer 344 rats (280–300 g) were used for the experiments (Envigo RMS, Inc., Oxford, MI, USA). All surgical procedures were performed under general anesthesia induced with 50 mg/kg of ketamine (Hospira, Inc., Lake Forest, IL, USA) and 5 mg/kg xylazine (Akorn, Inc., Lake Forest, IL, USA) intraperitoneally. After anesthesia, a 2 cm incision in a head-tail orientation with a size 15 scalpel blade was performed, and subsequently, four small separate subcutaneous pockets were created through tissue divulsion [40].

Square-shaped samples (10 mm height × 10 mm width × 1 mm depth) of electrospun GelMA+15%AZ fibers (scaffolds) were implanted. Antibiotic-free (i.e., pure) GelMA electrospun fibers and Bio-Gide^®^ (Geistlich Pharma North America Inc., Princeton, NJ, USA) were used as controls. After wound closure with Coated Vicryl^®^ polyglactin 910 (Ethicon Endo-Surgery, Inc., Cincinnati, OH, USA), the animals were allowed to recover from anesthesia. At 14- or 28-days post-implantation, the animals were euthanized by CO_2_ inhalation, and the implanted biomaterials with surrounding tissue were retrieved, fixed in 10% buffered formalin overnight, embedded in paraffin, cut into 6 μm-thick sections, and stained with hematoxylin and eosin (H&E) to investigate under light microscopy (Nikon E800, Nikon Corporation, Tokyo, Japan) for the presence of inflammatory cells and neovascularization. They were then, stained with picrosirius red (PSR) to analyze under polarized light microscopy the orientation, pattern, and interweaving of the collagen fibers in the implanted material, where greenish-yellow fibers were considered to be immature and thin, while yellowish-red fibers were considered to be mature and thick [41].

### 4.10. Statistical Analysis

All the analyses were performed with GraphPad Prism 9 software (GraphPad Software, San Diego, CA, USA). The fiber diameter and tensile strength were analyzed using a one-way analysis of variance. Each group’s inhibition zone and cytocompatibility data were compared using an analysis of variance that included a random effect to account for correlations within a specimen over time. Tukey’s post-hoc test was used to count for differences among groups. The level of significance was set at 5%.

## 5. Conclusions

The AZ-laden fibers (GelMA+15%AZ) have been shown to be a promising alternative for the sustained delivery of azithromycin for endodontic infection control, particularly within the context of regenerative endodontics due to their potent antimicrobial activity against *Aa* and *An* without compromising the biocompatible properties.

## Data Availability

The data presented in this study are available on request from the corresponding author.
